# The prognostic significance of electrocardiography findings in patients with coronavirus disease 2019: A retrospective study

**DOI:** 10.1002/clc.23628

**Published:** 2021-05-11

**Authors:** Deyan Yang, Jing Li, Peng Gao, Taibo Chen, Zhongwei Cheng, Kangan Cheng, Hua Deng, Quan Fang, Chunfeng Yi, Hongru Fan, Yonghong Wu, Liwei Li, Yong Fang, Guowei Tian, Wan Pan, Fan Zhang

**Affiliations:** ^1^ Department of Cardiology Peking Union Medical College Hospital, Chinese Academy of Medical Sciences & Peking Union Medical College Beijing China; ^2^ Department of Cardiology, Intervention Cardiology Center Wuhan No.1 Hospital, No.215 Zhongshan Avenue, QiaoKou District Wuhan China

**Keywords:** cardiac injury, coronavirus, electrocardiography, outcome

## Abstract

**Background:**

Coronavirus disease 2019 (COVID‐19) has reached a pandemic level. Cardiac injury is not uncommon among COVID‐19 patients. We sought to describe the electrocardiographic characteristics and to identify the prognostic significance of electrocardiography (ECG) findings of patients with COVID‐19.

**Hypothesis:**

ECG abnormality was associated with higher risk of death.

**Methods:**

Consecutive patients with laboratory‐confirmed COVID‐19 and definite in‐hospital outcome were retrospectively included. Demographic characteristics and clinical data were extracted from medical record. Initial ECGs at admission or during hospitalization were reviewed. A point‐based scoring system of abnormal ECG findings was formed, in which 1 point each was assigned for the presence of axis deviation, arrhythmias, atrioventricular block, conduction tissue disease, QTc interval prolongation, pathological Q wave, ST‐segment change, and T‐wave change. The association between abnormal ECG scores and in‐hospital mortality was assessed in multivariable Cox regression models.

**Results:**

A total of 306 patients (mean 62.84 ± 14.69 years old, 48.0% male) were included. T‐wave change (31.7%), QTc interval prolongation (30.1%), and arrhythmias (16.3%) were three most common found ECG abnormalities. 30 (9.80%) patients died during hospitalization. Abnormal ECG scores were significantly higher among non‐survivors (median 2 points vs 1 point, *p* < 0.001). The risk of in‐hospital death increased by a factor of 1.478 (HR 1.478, 95% CI 1.131–1.933, *p* = 0.004) after adjusted by age, comorbidities, cardiac injury and treatments.

**Conclusions:**

ECG abnormality was common in patients admitted for COVID‐19 and was associated with adverse in‐hospital outcome. In‐hospital mortality risk increased with increasing abnormal ECG scores.

## 1 INTRODUCTION

Since December 2019, coronavirus disease 2019 (COVID‐19) caused by the severe acute respiratory syndrome coronavirus 2 (SARS‐CoV‐2) quickly spread throughout the world and caused a global pandemic.^1^ Although clinical manifestations of COVID‐19 were mainly respiratory, cardiac injury, and arrhythmias were not uncommon and the association between cardiac injury and poor in‐hospital outcome had been determined.^2^, ^3^ As a simple and easily obtainable tool to identify patients with acute or chronic cardiac disease, ECG is frequently performed in patients with cardiovascular disease and was also studied in pneumonia,^4^ severe acute respiratory syndrome (SARS),^5^ and Middle East respiratory syndrome (MERS).^6^ However, systematic studies of ECG characteristics in COVID‐19 patients were limited^7^, ^8^ and its prognostic significance remained to be fully elucidated.^9^

The purpose of this study was to describe the electrocardiographic characteristics and to identify the prognostic significance of ECG findings at admission or during hospitalization in patients with COVID‐19.

## 2 METHODS

### 2.1 Study design and patient selection

In this single center, observational study, consecutive patients with laboratory SARS‐Cov‐2 RNA detection confirmed COVID‐19 and definite in‐hospital outcome admitted in Wuhan No.1 Hospital, China from 25 December, 2019 to 10 March, 2020 were retrospectively included. Wuhan No.1 Hospital, China is a designated hospital to treat patients with COVID‐19. Throat‐swab specimens were obtained for SARS‐CoV‐2 detection from suspected patients using real‐time polymerase chain reaction (PCR) assay, which was performed by local Centers for Disease Control and Prevention and Wuhan No.1 Hospital, China. This study was approved by the institutional ethics board of Wuhan No.1 Hospital, China and complies with the Declaration of Helsinki. Consent was obtained from patients or patients' family member.

### 2.2 Clinical data collection

Demographic information (age and sex) and clinical data consisting of disease duration, blood pressure at admission, comorbidities, serum level of high‐sensitive cardiac troponin I (hs‐cTnI), and treatment data were extracted from medical record. Disease duration was defined as time from symptom onset to admission. Cardiac injury was diagnosed if serum level of hs‐cTnI was above the 99th percentile upper reference limit (which was 0.026ug/L in our hospital).

### 2.3 12‐Lead electrocardiography

Resting standard 12‐lead ECGs were performed by trained physicians using PageWriter TC10 (Philips, Amsterdam, Noord‐Holland, Netherland) machine or MAC 800 (GE Healthcare, Chicago, IL) machine in all patients at admission or during hospitalization using a paper speed of 25 mm/s and a sensitivity of 1 mV = 10 mm. The initial ECG record of each patient was reviewed. Heart rate, PR interval, QTc interval (corrected by Bazett's formula) and mean frontal plane QRS electrical axis were measured automatically by the ECG machine/computer‐generated measurements. ECG parameters (definitions listed in the Table S1) were reviewed and confirmed by the principal investigators (D.Y. and J.L.). Discordances were solved by consensus, with the supervision of the senior expert (F.Z.) in electrocardiography.

Patients were considered to have arrhythmias if they had sinus tachycardia, sinus bradycardia, sinus node arrest, or atrial fibrillation and were considered to have conduction tissue disease if they had right bundle branch block (RBBB), left bundle branch block (LBBB), or left anterior fascicular block(LAFB).^10^ ST‐segment change included ST‐elevation and ST‐depression and T‐wave change was consisted of inverted T‐wave and flat T‐wave (Figure S1A‐S1B). To evaluate the prognostic significance of abnormal ECG findings, we created a point‐based scoring system, in which 1 point each was assigned for the presence of axis deviation (Figure S1C‐S1D), arrhythmias, atrioventricular block, conduction tissue disease, QTc interval prolongation, pathological Q wave, ST‐segment change, and T‐wave change. We calculated abnormal ECG scores by adding 1 point each for any of the eight ECG findings aforementioned. Such ECG scoring was analyzed as a continuous variable.

### 2.4 In‐hospital outcome

All patients were followed up during hospitalization. The in‐hospital outcome comprised incidence of in‐hospital death or discharge. Patients were discharged if they had relieved clinical symptoms, normal body temperature, significant resolution of inflammation as shown by chest radiography, and at least 2 consecutive negative results shown by real‐time PCR assay for COVID‐19.^11^ The vital status of patients was determined by medical record. Discharged patients were censored at the date of discharge.

### 2.5 Statistical analysis

Continuous variables are presented as means with standard deviations (SD) for normally distributed data or median with interquartile range (IQR) for non‐normal distribution. Normal distribution of variables was assessed by the Kolmogorov–Smirnov test. Categorical variables were expressed as frequencies and percentages. Patients were categorized as non‐survivors versus survivors. Student's *t* test or the Mann–Whitney test was used to test intergroup differences in continuous variables where appropriate. Differences in categorical variables were tested by Fishers' exact test. Univariable and multivariable Cox regression were used to analyze the association between baseline variables and in‐hospital death. Variables that were significantly associated with in‐hospital death (*p* < 0.05) in the univariable analysis were retained in the multivariable model (forward stepwise likelihood ratio selection method).

To evaluate the association between abnormal ECG and in‐hospital death, we established two Cox regression models. In model 1, univariable and multivariable Cox regression were firstly performed on variables including age, comorbidities, cardiac injury and abnormal ECG. Secondly, variables consisting of treatment with glucocorticoid, immune globulin, and mechanical ventilation were added to the identified independent variables in model 1 to assess whether they maintained a significant association with in‐hospital mortality (model 2). Time to in‐hospital death was illustrated by the Kaplan–Meier method and log‐rank trend test was used to compared the survival curve between patients with or without abnormal ECG point(s). The statistical analysis was performed in SPSS (version 19.0). *p*‐values <0.05 (2‐sided) were considered statistically significant.

## 3 RESULTS

### 3.1 Patient characteristics

A total of 319 patients with laboratory confirmed COVID‐19 were admitted in our hospital from 25 December, 2019 to 10 March, 2020. 13 (4.1%) patients who were transferred to other hospital without definite in‐hospital endpoint were excluded. Hence, the remaining 306 patients were finally included in the present study. The mean age of this cohort was 62.84 ± 14.69 years old and 147 (48.0%) were male. A total of 157 patients (51.3%) were with comorbidities. The proportion of hypertension, diabetes, coronary artery disease, previous stroke, atrial fibrillation history, chronic kidney disease, and chronic obstructive pulmonary disease was 41.8% (128 patients), 16.7% (51 patients), 12.1% (37 patients), 7.8% (24 patients), 0.7% (2 patients), 5.2% (16 patients), and 3.9% (12 patients), respectively. The proportion of cardiac injury was 14.4% (44 patients) and the median serum level of hs‐cTnI was 0.005 (range 0.000–4.700, IQR 0.001,0.012) μg/L.

Compared with survivors, non‐survivors were older (mean age 79.17 ± 8.80 vs 61.07 ± 14.10, *p* < 0.001) and had a higher proportion of comorbidities (83.3% vs 47.8%, *p* < 0.001) and more likely to have cardiac injury (70.0% vs 8.3%, *p* < 0.001). The median serum hs‐cTnI level was also higher among non‐survivor group (0.048 [0.023,0.174] vs 0.004 [0.001,0.009] μg/L, *p* < 0.001). Non‐survivors had significantly faster heart rate. The proportion of sex, disease duration, and blood pressure at admission of the two groups were similar. Baseline demographic and clinical characteristics were listed in Table 1.

**TABLE 1:** Comparison of demographics, clinical characteristics and treatments between non‐survivors versus survivors

Variables	All patients (*n* = 306)	Non‐survivors (*n* = 30)	Survivors (*n* = 276)	*p*‐value
Demographics characteristics				
Male – *n* (%)	147 (48%)	19 (63%)	128 (46%)	0.086
Age, years	63 ± 15	79 ± 9	61 ± 14	**<0.001**
Clinical characteristics				
Disease duration, days	7 (5–12)	8 (5–10)	7 (5–12)	0.799
Heart rate	80 (70–93)	95 (83–106)	78 (70–91)	**<0.001**
SBP on admission, mmHg	130 (120–140)	131 (120–144)	130 (120–140)	0.674
DBP on admission, mmHg	79 (71–85)	80 (69–87)	79 (71–85)	0.854
Comorbidities – *n* (%)	157 (51%)	25 (83%)	132 (48%)	**<0.001**
Hypertension – *n* (%)	128 (42%)	21 (70%)	107 (39%)	**0.001**
Diabetes – *n* (%)	51 (17%)	8 (27%)	43 (16%)	0.126
CAD – *n* (%)	37 (12%)	10 (33%)	27 (10%)	**0.001**
Previous stroke – *n* (%)	24(8%)	8(27%)	16(6%)	**0.001**
AF history – *n* (%)	2 (0.7%)	1 (3%)	1 (0.4%)	0.187
CKD – *n* (%)	16 (5%)	7 (23%)	9 (3%)	**0.001**
COPD – *n* (%)	12 (4%)	5 (17%)	7 (3%)	**0.003**
hs‐cTnI (μg/L)	0.005 (0.001–0.012)	0.048 (0.023–0.174)	0.004 (0.001–0.009)	**<0.001**
Cardiac injury – *n* (%)	44 (14%)	21 (70%)	23 (8%)	**<0.001**
Hospital stay, days	22 (13–29)	12 (6–18)	23 (14–30)	**<0.001**
Treatment				
Antivirus – *n* (%)	301 (98%)	29 (97%)	272 (99%)	0.405
Glucocorticoid – *n* (%)	102 (33%)	27 (90%)	75 (27%)	**<0.001**
Immune globulin – *n* (%)	37 (12%)	14 (47%)	23 (8%)	**<0.001**
Chloroquine – *n* (%)	18 (6%)	2 (7%)	16 (6%)	0.692
Mechanical ventilation – *n* (%)	7 (2%)	6 (20%)	1 (0.4%)	**<0.001**

*Note*: Statistically significant *p*‐values (*p* < 0.05) are shown in bold.

Abbreviations: AF, atrial fibrillation; CAD, coronary artery disease; CKD, chronic kidney disease; COPD, chronic obstructive pulmonary disease; DBP, diastolic blood pressure; hs‐cTnI, high‐sensitive cardiac troponin I; SBP, systolic blood pressure.

Overall, majority of patients were treated with antivirus (301 patients, 98.4%). The proportion of arbidol, ganciclovir, oseltamivir, lopinavir/ritonavir (Kaletra), and remdesivir was 90.8% (278 patients), 16.3% (50 patients), 16.3% (50 patients), 3.9% (12 patients), and 1.3% (4 patients), respectively. The proportion of patients treated with glucocorticoid, immune globulin and chloroquine was 33.3% (102 patients), 12.1% (37 patients), and 5.9% (18 patients), respectively. Mechanical ventilation was used in 7 patients (2.3%). A total of 223 patients (72.9%) were on Moxifloxacin. Compared with survivors, non‐survivors presented with a significantly higher proportion of glucocorticoid (90.0% vs 27.2%, *p* < 0.001), immune globin (46.7% vs 8.3%, *p* < 0.001), and mechanical ventilation therapy (20.0% vs 0.4%, *p* < 0.001). Treatment data were depicted in Table 1.

### 3.2 ECG findings

**FIGURE 1:**
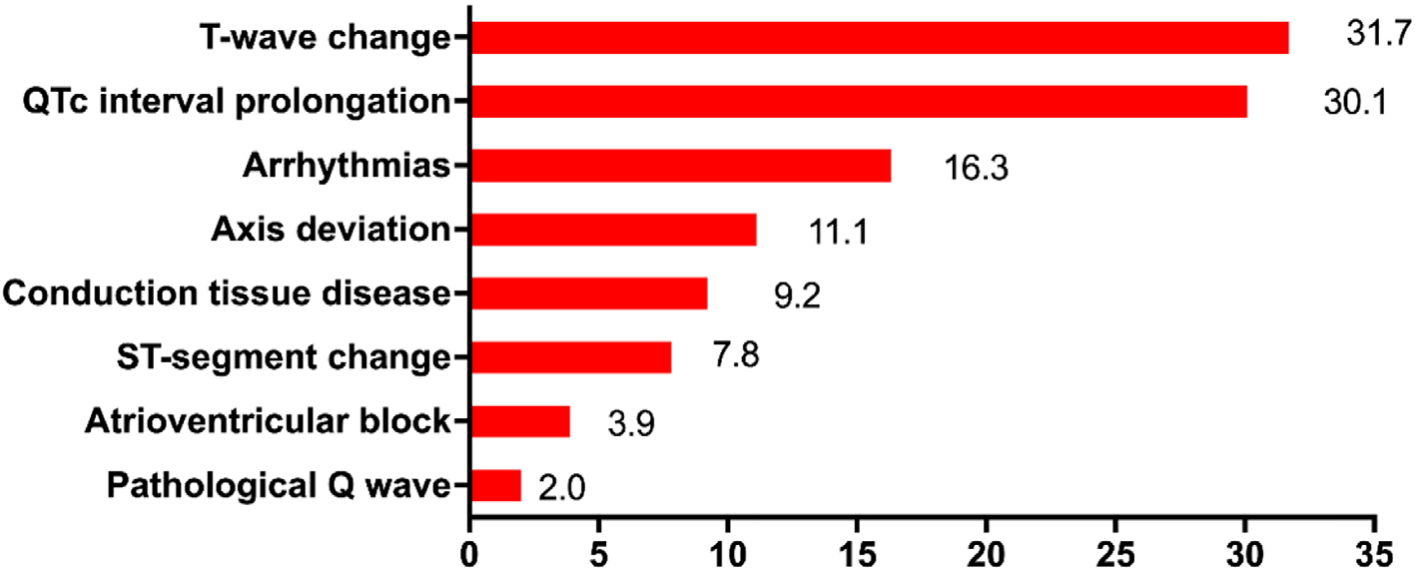
Abnormal electrocardiography findings (%) of 306 patients of with coronavirus disease 2019

Initial ECGs were performed at admission (within 48 h) in 274 (89.5%) patients. The remaining 32 (10.5%) patients underwent ECGs examination during hospitalization with median 14 (8.3,18.0) days after admission. As shown in Table 2 and Figure 1, T‐wave change (97 patients, 31.7%) was the most common abnormal finding and QTc interval prolongation, arrhythmias, axis deviation, conduction tissue disease, ST‐segment change, atrioventricular block, and pathological Q wave were present in 92 patients (30.1%), 50 patients (16.3%), 34 patients (11.1%), 28 patients (9.2%), 24 patients (7.8%), 12 patients (3.9%), and 6 patients (2.0%), respectively.

**TABLE 2:** Comparison of ECG findings between non‐survivors versus survivors

Variables	All patients (*n* = 306)	Non‐survivors (*n* = 30)	Survivors (*n* = 276)	*p*‐value
Axis deviation – *n* (%)	34 (11%)	10 (33%)	24 (9%)	**<0.001**
Right‐axis deviation‐*n* (%)	7 (2%)	3 (10%)	4 (1%)	**0.023**
Left‐axis deviation –*n* (%)	26 (9%)	6 (20%)	20 (7%)	**0.030**
Extremely axis deviation – *n* (%)	1 (0.3%)	1 (3%)	0 (0%)	0.098
Arrhythmias – *n* (%)	50 (16%)	12 (40%)	38 (14%)	**0.001**
Sinus tachycardia –*n* (%)	29 (10%)	7 (23%)	22 (8%)	**0.014**
Sinus bradycardia –*n* (%)	10 (3%)	0 (0%)	10 (4%)	0.606
Sinus node arrest –*n* (%)	1 (0.3%)	1 (3%)	0 (0%)	0.098
AF –*n* (%)	10 (3%)	4 (13%)	6 (2%)	**0.010**
AVB – *n* (%)	12 (4%)	3 (10%)	9 (3%)	0.102
First degree AVB – *n* (%)	10 (3%)	2 (7%)	8 (3%)	0.256
Second degree AVB Mobitz type I – *n* (%)	2 (0.7%)	1 (3%)	1 (0.4%)	0.187
CTD –*n* (%)	28 (9%)	6 (20%)	22 (8%)	**0.042**
RBBB – *n* (%)	20 (7%)	5 (17%)	15 (5%)	**0.035**
LBBB – *n* (%)	1 (0.3%)	0 (0%)	1 (0.4%)	>0.999
LAFB – *n* (%)	10 (3%)	2 (7%)	8 (3%)	0.256
QTc interval	440 (422–459)	467 (428–479)	439 (421–457)	**0.002**
QTc interval prolongation –*n* (%)	92 (30%)	18 (60%)	74 (27%)	**0.001**
PR interval^a^	154 (140–166)	160 (133–177)	154 (140–166)	0.521
Pathological Q wave–*n* (%)	6 (2%)	1 (3%)	5 (2%)	0.464
ST‐segment change – *n* (%)	24 (8%)	8 (27%)	16 (6%)	**0.001**
ST‐segment elevation–*n* (%)	1 (0.3%)	0 (0%)	1 (0.4%)	>0.999
ST‐segment depression–*n* (%)	23 (8%)	8 (27%)	15 (5%)	**0.001**
T‐wave change – *n* (%)	97 (32%)	18 (60%)	79 (29%)	**0.001**
Inverted T‐wave – *n* (%)	22 (7%)	4 (13%)	18 (7%)	0.251
Flat T‐wave – *n* (%)	75 (25%)	14 (47%)	61 (22%)	**0.006**
Abnormal ECG point(s)	1 (0–2)	2 (1–4)	1 (0–2)	**<0.001**

*Note*: Statistically significant p‐values (*p* < 0.05) are shown in bold.

Abbreviations: AF, atrial fibrillation; AVB, atrioventricular block; CTD, conduction tissue disease; LAFB, left anterior fascicular block; LBBB, left bundle branch block; RBBB, right bundle branch block.

^a^ PR interval was compared between non‐survivors (*n* = 25) and survivors (*n* = 270), who were in sinus rhythm.

As listed in Table 2, non‐survivors had significantly longer QTc interval and higher rate of axis deviation, arrhythmias, conduction tissue disease, QTc interval prolongation, ST‐segment change, and T‐wave change. The PR interval and the proportion of atrioventricular block and pathological Q wave of the two groups were similar. Atrial fibrillation and sinus tachycardia were detected in 10 (3.3%) and 29 (9.5%) patients respectively and both of them were more common in non‐survivors. First degree atrioventricular block (AVB) was seen in 10 patients (3.3%) and second degree AVB Mobitz type I was found in 2 patients (0.7%). The proportion of right bundle branch block was higher among non‐survivors but left bundle branch block was not. Among patients with ST‐segment change, the majority of them developed ST‐segment depression (95.8%. 23/24) and only one patient developed ST‐segment elevation. The proportion of flat T‐wave was higher among non‐survivors but inverted T‐wave was not.

**FIGURE 2:**
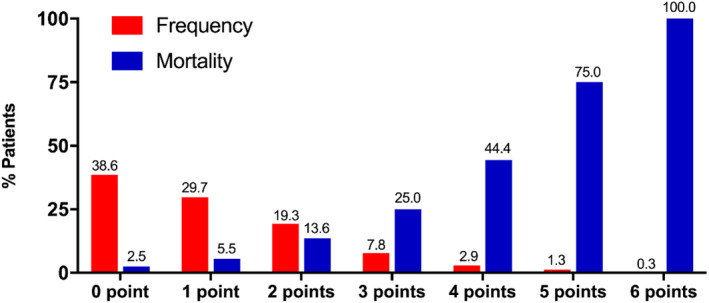
Frequency and mortality of patients with abnormal electrocardiography points from 0 to 6

As shown in Figure 2, the proportion of abnormal ECG score from 0 point to 6 points was 38.6% (118 patients), 29.7% (91 patients), 19.3% (59 patients), 7.8% (24 patients), 2.9% (9 patients), 1.3% (4 patients), and 0.3% (1 patient), respectively. No patient had abnormal ECG score of 7 or 8 points. The median abnormal ECG score was significantly higher in non‐survivor group (2.0[1.0,4.0] vs 1.0[0.0,2.0], *p* < 0.001). Table 2 summarized the electrocardiographic characteristics in detail.

### 3.3 Association between ECG findings and in‐hospital mortality

**FIGURE 3:**
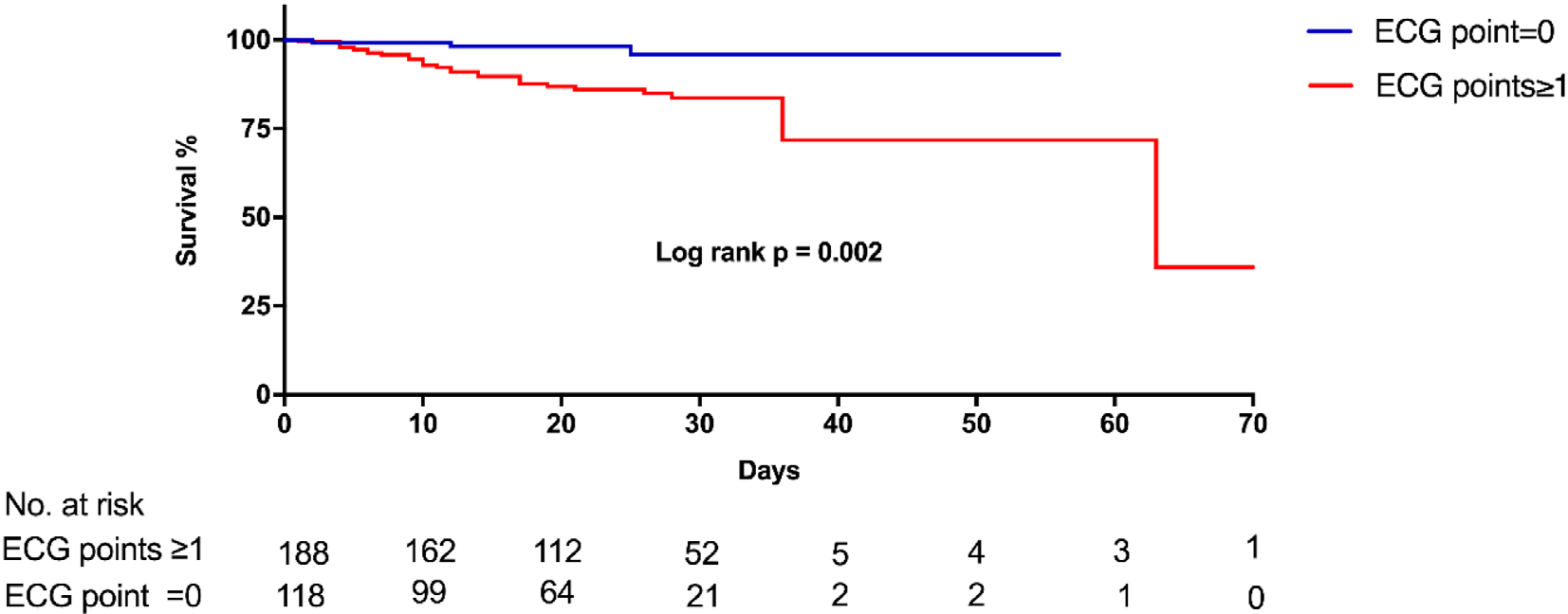
Kaplan–Meier curves showing in‐hospital mortality according to abnormal electrocardiography point(s)

The media duration of in‐hospital stay was 22.00 (IQR, 12.75,29.00) days in all patients and was significantly shorter in non‐survivors group (11.50 [IQR, 6.00,17.50] days vs 23.00 [IQR, 14.00,30.00] days, *p* < 0.001). The media duration of in‐hospital stay was similar between patients with or without abnormal ECG point(s) (23.00 [IQR, 13.00,30.00] days vs 21.00 [IQR, 12.00,28.00] days, *p* = 0.193). During in‐hospital follow‐up, a total of 30 patients (9.8%) died and 276 patients (90.2%) were cured and discharged. As shown in Figure 2, the in‐hospital mortality rate was 2.5% (3/118), 5.5% (5/91), 13.6% (8/59), 25.0% (6/24), 44.4% (4/9), 75.0% (3/4), and 100.0% (1/1) in patients with abnormal ECG points of from 0 to 6, respectively. Compared with patients without abnormal ECG point (0 point), the in‐hospital mortality rate was higher among patients with abnormal ECG point(s) (≥1 points) (14.4% vs 2.5%, *p* = 0.001). As shown in the Kaplan–Meier survival curves in Figure 3, patients with abnormal ECG point(s) had more and earlier hospital‐death (Log‐rank *p* = 0.002).

In multivariable Cox regression model 1, age, cardiac injury and abnormal ECG were associated with in‐hospital mortality. After adjusting for age, cardiac injury, glucocorticoid treatment, immune globin treatment, and mechanical ventilation, the multivariable Cox regression model 2 showed that in‐hospital mortality was associated with abnormal ECG points (HR 1.478, 95% CI 1.131–1.933, per one point increase, *p* = 0.004) and age (HR 1.054, 95%CI 1.011–1.100, per year increase, *p* = 0.014), cardiac injury (HR 4.905, 95%CI 1.985–12.116, *p* = 0.001), and glucocorticoid treatment (HR 10.330, 95%CI 3.095–34.475, *p* < 0.001) were also independently associated with in‐hospital mortality (Table 3).

**TABLE 3:** Univariable and multivariable Cox regression analysis

	Univariable regression analysis			Multivariable regression analysis		
Model 1	HR	95% CI	*p*‐value	HR	95% CI	*p*‐value
Age	1.111	1.071–1.153	**<0.001**	1.062	1.021–1.105	**0.003**
Comorbidities	4.128	1.572–10.837	**0.004**			
Cardiac injury	15.620	7.114–34.295	**<0.001**	5.668	2.359–13.616	**<0.001**
Abnormal ECG	1.978	1.614–2.425	**<0.001**	1.479	1.121–1.950	**0.006**

	Univariable regression analysis			Multivariable regression analysis		
Model 2	HR	95% CI	*p*‐value	HR	95% CI	*p*‐value
Age	1.111	1.071–1.153	**<0.001**	1.054	1.011–1.100	**0.014**
Cardiac injury	15.620	7.114–34.295	**<0.001**	4.905	1.985–12.116	**0.001**
Abnormal ECG	1.978	1.614–2.425	**<0.001**	1.478	1.131–1.933	**0.004**
Glucocorticoid	17.784	5.376–58.833	**<0.001**	10.330	3.095–34.475	**<0.001**
Immune globulin	6.068	2.928–12.577	**<0.001**			
MV	12.744	5.154–31.507	**<0.001**			

*Note*: Statistically significant *p*‐values (*p* < 0.05) are shown in bold.

Abbreviations: CI, confidence interval; DBP, diastolic blood pressure; HR, hazard ratio; MV, mechanical ventilation; SBP, systolic blood pressure.

## 4 DISCUSSION

The main findings of our study are threefold: (a) Various abnormal ECG findings were common among patients with COVID‐19; (b) Proportions of many ECG abnormalities were significantly higher in non‐survivors; (c) Abnormal ECG was significantly associated with in‐hospital mortality in COVID‐19 patients after adjusting for potential confounding factors. Furthermore, per one point of abnormal ECG increasing was associated with a 47.8% increase in the relative risk of in‐hospital mortality.

Abnormal ECGs were frequently encountered among COVID‐19 patients. A recent study from Italy including 324 patients with COVID‐19 had found that any abnormal finding on the ECG was found in 120 patients (37%)^9^ . The proportion of abnormal ECG was as high as 93% among critical ill COVID‐19 patients.^8^ Nearly two third of patients in our study had at least one point of ECG abnormalities scoring. Although ECG data of patients with pneumonia caused by non‐SARS‐CoV‐2 pathogens was unavailable in our study, previous study had shown that ECG abnormalities prevalence were similar between COVID‐19 and acute infectious respiratory disease caused by other pathogens (37.0% vs 43.5%, *p* = 0.13) and no differences in ECG abnormalities were found between the COVID‐19 group and no‐COVID‐19 group.^12^

Lanza GA et al had reported that atrial fibrillation, increasing heart rate, a QRS duration ≥110 ms (including patients with LBBB or RBBB) and ST‐segment depression were associated with short term mortality of hospitalized COVID‐19 patients in univariable Cox regression analysis.^9^ The relation between abnormal ECG findings and in‐hospital death of our present study was consistent with Lanza GA's study. We speculated that both of atrial fibrillation and increasing heart rate might result from hypoxia, fever and hyperinflammatory and ST segment change might be due to myocardial ischemia or injury. Bundle branch block and QRS lengthening indicated a delay in ventricular depolarization which might be explained by myocardial injury. Prevalence of incomplete RBBB and complete RBBB was 9% and 11% respectively in a large series of critically ill COVID‐19 patients and the interpretation of such ECG abnormalities proposed by Matteo Bertini et al was right ventricular pressure overload.^8^ Due to the limit data of echocardiography in the present study, we did not explore the pathophysiological insights of RBBB.

A smaller series^13^ including 50 COVID‐19 patients had found that ST‐T abnormalities were common (30%) at admission, which was comparable with our data. In a study of 107 patients hospitalized with COVID‐19, first‐degree AVB was seen in 20 (18.7%) patients and 1 (0.9%) patient developed transient Mobitz II AVB.^7^ First degree and second degree AVB were also seen in our study. This might suggest that conduction system could be involved by SARS‐CoV‐2 infection. The proportion of QTc interval prolongation was 30% in our study, which was in agreement with previous report with corresponding proportion of 38%.^8^ QTc interval prolongation might be attributed to serious illness and hypoxemia.^8^ As Moxifloxacin and Chloroquine was used in 73% and 6% in the present series, respectively, an alternative explanation might be medications potentially affecting QTc interval.

To the best of our knowledge, no study has so far evaluated the prevalence and clinical implications of QRS axis deviation in COVID‐19 patients. Axis deviation was detected in 11% of patients in our study and was more often among non‐survivors (33% vs 9%, *p*<0.001). Although the precise mechanism of axis deviation was largely unknown, axis deviation might be attributed to depolarization disorder, which could be seen in pulmonary hypertension, left ventricular hypertrophy and myocardial ischemia.

Cardiac injury defined as hs‐cTnI elevation was common in COVID‐19 patient and had been determined to be associated with mortality.^2^, ^3^ Shi S et al reported that 19.7% of COVID‐19 patients had cardiac injury and patients with cardiac injury were at a higher risk of death.^3^ In our present study, a total of 44 patients (14.4%) had cardiac injury and cardiac injury was independently associated with in‐hospitalized mortality, which was in line with previous studies. A recent study including 324 patients affected by COVID‐19 had found that abnormal ECG was significantly associated with death when adjusted for cTnI levels in a minority of patients with available cTnI data.^9^ Consistent with this finding, abnormal ECG showed significant association with mortality after adjusting for cardiac injury and other relevant clinical and treatment variables in our large series of 306 patients with cTnI measurement.

Importantly, our data indicated that relative risk of in‐hospital mortality increased with increasing abnormal ECG scores. Abnormal ECG scores in our study reflected a wide spectrum of cardiac involvement including arrhythmias, conduction disorders and ventricular depolarization and repolarization disorders. The mechanisms of cardiovascular manifestations in COVID‐19 patients remained unclear and several putative mechanisms had been proposed such as direct viral myocardial injury, stress cardiomyopathy, acute coronary syndrome, oxygen supply and demand mismatch and systemic hyperinflammatory response.^14^ The increase of abnormal ECG scores might be attributed to severer acute cardiac complications of COVID‐19 induced by multiple mechanisms and/or more pre‐existing cardiovascular diseases.

Complications and poor outcomes more frequently occurred in elderly COVID‐19 patients.^11^, ^15^ Consistent with these findings, increasing age was independently associated with in‐hospital death in the present study. An unexpected finding was that treatment with glucocorticoid was also related to mortality in multivariable Cox regression analysis. A possible explanation might be that severer patients tended to receive glucocorticoid therapy^15^ and non‐survivors also was with a higher proportion of glucocorticoid therapy.^11^

## 5 CONCLUSIONS

Our data show that ECG abnormality was common among admitted patients for COVID‐19 and was associated with adverse in‐hospital outcome. In‐hospital mortality risk increased with increasing abnormal ECG scores, suggesting that close observation should be kept on patients with multiple ECG abnormalities during their hospitalization. ECG might be an easy tool for risk stratification in such patients.

## 6 LIMITATION

Some limitations of our study should be acknowledged. Firstly, only initial ECGs on admission or during hospitalization were reviewed. Neither previous ECGs nor subsequent ECGs were analyzed for comparison. It is difficult to distinguish pre‐existing cardiac disease between acute infective status related ECG abnormalities. Secondly, echocardiographic data was available in only a minority of patients and was not included in our analysis. Thirdly, the follow‐up of patients was short and our results might not be representative of the long‐term prognosis. Fourthly, the event rate was low and the multivariable Cox regression analysis with many variates may be less reliable. Finally, several individual ECG abnormalities were detected in small numbers of patients and the estimate of their prognostic role should be further verified in larger populations.

## CONFLICT OF INTEREST

All authors declare no conflicts of interest that might be relevant to the contents of this manuscript.

## Supporting information


**Table S1** Definition of the included ECG parameters

**Figure S1 (A)** An ECG of an 80‐year old female shown inverted T‐wave in leads II, III, aVF, V2 to V6 and a prolonged PR interval was also seen. **(B)** An ECG of a 56‐year old female shown flat T‐wave in leads V2 to V6. **(C)** An ECG of a 82‐year old female shown left‐axis deviation (QRS axis − 61°). Atrial fibrillation and intraventricular conduction abnormality could also be seen. **(D)** An ECG of a 66‐year old female shown right‐axis deviation (QRS axis 133°). Sinus node arrest with junctional escape rhythm could also be seen in this case.

## Data Availability

The data that support the findings of this study are available on request from the corresponding author. The data are not publicly available due to privacy or ethical restrictions.
