# Supplementary material for: The prognostic significance of electrocardiography findings in patients with coronavirus disease 2019: A retrospective study
Source: Clin Cardiol. 2021 May 11;44(7):963–70. doi: 10.1002/clc.23628 (PMC8237010; doi:10.1002/clc.23628)
Supplement: Supplementary file 1 — Table S1 Definition of the included ECG parameters [file CLC-44-963-s002.docx]

**Supplemental Table 1**

Definition of the included ECG parameters

| Variable | Definition |
| --- | --- |
| Right-axis deviation | Mean frontal plane QRS axis +90° to ±180° |
| Left-axis deviation | Mean frontal plane QRS axis equal or leftward of -30° to -90° |
| Extremely axis deviation | Mean frontal plane QRS axis -90° to ±180° |
| Sinus tachycardia | Sinus rhythm with heart rate above 100 beat per minute |
| Sinus bradycardia | Sinus rhythm with heart rate below 60 beat per minute |
| Sinus node arrest | No evidence of sinus node depolarization |
| Atrial fibrillation | Absolutely irregular RR intervals and no discernible, distinct P waves |
| First-degree AVB | P waves associated with 1:1 atrioventricular conduction and a PR interval >200 ms |
| Second-degree AVB Mobitz type I | P waves with a constant rate (<100 bpm) with a periodic single nonconducted P wave associated with P waves before and after the non-conducted P wave with inconstant PR intervals |
| RBBB | rsr′, rsR′, rSR′, or rarely a qR in leads V1 or V2. The R′ or r′ deﬂection is usually wider than the initial R wave. S wave of greater duration than R wave or >40 ms in leads I and V6. Normal R peak time in leads V5 and V6 but >50 ms in lead V1. |
| LBBB | Broad notched or slurred R wave in leads I, aVL, V5, and V6 and an occasional RS pattern in V5 and V6 attributed to displaced transition of QRS complex. Absent Q waves in leads I, V5, and V6, but in the lead aVL, a narrow Q wave may be present in the absence of myocardial pathology. R peak time >60 ms in leads V5 and V6 but normal in leads V1, V2, and V3, when small initial R waves can be discerned in the precordial leads |
| LAFB | QRS duration <120 ms. Frontal plane axis between -45° and -90°. qR (small r, tall R) pattern in lead aVL. R-peak time in lead aVL of ≥45 ms. rS pattern (small r, deep S) in leads II, III, and aVF |
| QTc prolongation | QTc interval≥450ms in male or QTc interval ≥460ms in female |
| Pathological Q wave | Negative deflection preceding R-wave with duration ≥40 ms or ≥2 mV deep or ≥ 25% of QRS amplitude |
| ST-elevation | ≥0.2 mV elevation of the ST-segment measured at the J-point in leads V2 and V3 and ≥0.1 mV in any other two anatomically consecutive leads |
| ST-depression | ≥0.05mV 60ms after the J-point in leads V2 and V3 and ≥0.1 mV in any other two anatomically consecutive leads |
| Inverted T-wave | Negative T-wave with amplitude≥0.1mV in leads I, II, aVL, and V2 to V6 |
| Flat T-wave | Peak T-wave amplitude is between 0.1 and -0.1 mV in leads I, II, aVL (with an R wave taller than 0.3 mV), and V4 to V6 |

Abbreviations: AVB = atrioventricular block; RBBB = right bundle branch block; LBBB = left bundle branch block; LAFB = left anterior fascicular block.
