# Supplementary figures and images for: The prognostic significance of electrocardiography findings in patients with coronavirus disease 2019: A retrospective study
Source: Clin Cardiol. 2021 May 11;44(7):963–70. doi: 10.1002/clc.23628 (PMC8237010; doi:10.1002/clc.23628)

**Figure S1**

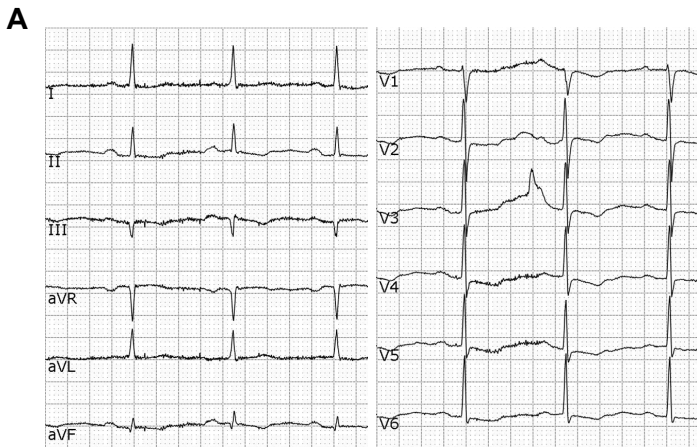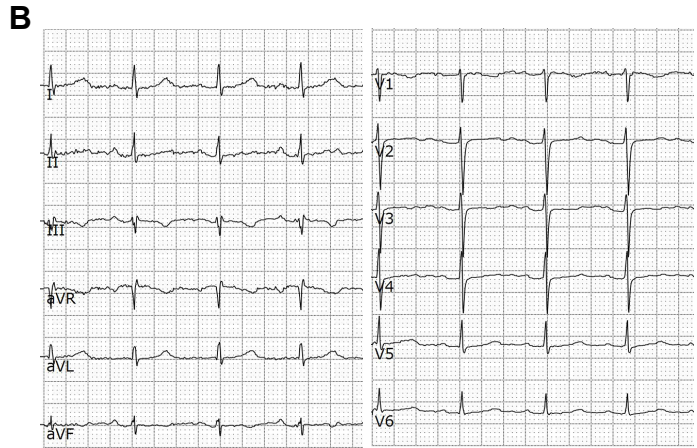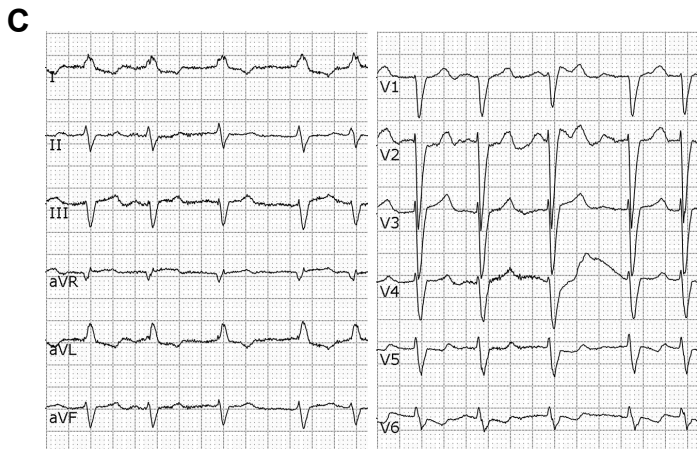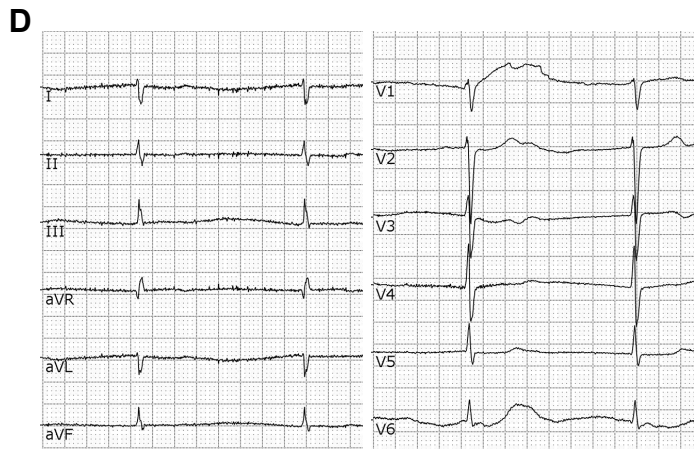

Supplement: Supplementary file 2 — Figure S1 (A) An ECG of an 80‐year old female shown inverted T‐wave in leads II, III, aVF, V2 to V6 and a prolonged PR interval was also seen. (B) An ECG of a 56‐year old female shown flat T‐wave in leads V2 to V6. (C) An ECG of a 82‐year old female shown left‐axis deviation (QRS axis − 61°). Atrial fibrillation and intraventricular conduction abnormality could also be seen. (D) An ECG of a 66‐year old female shown right‐axis deviation (QRS axis 133°). Sinus node arrest with junctional escape rhythm could also be seen in this case. [file CLC-44-963-s001.pdf]
